# Physical activity program for patients with dementia and their relative caregivers: randomized clinical trial in Primary Health Care (AFISDEMyF study)

**DOI:** 10.1186/1471-2377-14-63

**Published:** 2014-04-01

**Authors:** Emiliano Rodriguez-Sánchez, José María Criado-Gutiérrez, Sara Mora-Simón, M Paz Muriel-Diaz, Manuel A Gómez-Marcos, José I Recio-Rodríguez, M Carmen Patino-Alonso, Luis F Valero-Juan, José A Maderuelo-Fernandez, Luis García-Ortiz

**Affiliations:** 1 Primary Health Care Research Unit The Alamedilla. The Alamedilla Primary Health Center, Castilla-Leon Health Service–SACYL, IBSAL, Salamanca, Spain; 2 Medicine Department, University of Salamanca, Salamanca, Spain; 3 Department of Physiology and Pharmacology Department, University of Salamanca, Salamanca, Spain; 4 Basic Psychology, Psychobiology and Behavioral Sciences Methodology Department, University of Salamanca, Salamanca, Spain; 5 Statistics Department, University of Salamanca, Salamanca, Spain; 6 Preventive Medicine, Public Health and Medical Microbiology Department, University of Salamanca, Salamanca, Spain; 7 Unidad de Investigación, Centro de Salud La Alamedilla, 37003 Salamanca, Spain

**Keywords:** Dementia, Caregivers, Physical activity, Pedometer, Cardiovascular risk

## Abstract

**Background:**

The aging of the population has led to the increase of chronic diseases, especially dementia and cardiovascular diseases, and it has become necessary for their relatives to dedicate more time in caregiving.

The objective in the first phase of this study is to evaluate the effectiveness of a Primary Health Care procedure to increase the physical activity of people with dementia and their relative caregivers. Also the effect on the cognitive state and cardiovascular risk will be assessed.

**Methods/Design:**

Design: Clinical, multicentric and randomized trial. A simple random sampling to select 134 patients diagnosed with dementia will be carried out. After contacting their relatives, his/her participation in the trial will be requested. A basal assessment will be made and the participants will be asigned to control or intervention group (1:1). Variables: The main measure will be the assessment of physical activity (podometer and 7-PAR) in patients and caregivers. In patients with dementia: ADAS-cog, functional degree and cardiovascular risk. In caregivers: cardiovascular risk, general health and quality of life. **Intervention**: For 3 months, participants will receive instructions to do physical activity with an adapted program. This program will be designed and applied by Primary Health Care professionals in patients with dementia and their caregivers. The control group will receive regular care. **Analysis:** An intention-to-treat analysis will be carried out by comparing the observed differences between basal, 6 and 12 months measures. Change in the mean of daily steps assessed with the podometer and 7-PAR will be the main result.

**Discussion:**

If the main hypothesis is confirmed, it could be useful to improve the cognitive state of patients with dementia, as well as the cardiovascular risk of all of them. The results can be good to improve technical features of the devices that register the physical activity in the patients with dementia, and it could facilitate its commercialization.

**Trial registration:**

Clinical Trials.gov Identifier: NCT02044887.

## Background

Different types of dementia and cardiovascular diseases (CVD) are the most important health problems in elderly people. In Spain, ischemic heart disease is the main cause of losing years of life adjusted by disability in men, while the main cause in women is dementia [1]. The aging of the population has led to an increase in chronic diseases and disabilities. It has become necessary for relatives to dedicate more time in caregiving, especially women. This makes it a requirement to incorporate a gender perspective into the contents of this research about these diseases. For all these reasons it is important to find strategies to help patient with dementia (PWD) or with CVD and their relative caregivers (RC).

### Treatment of people with dementia

Nowadays it is recommended to approach the treatment of dementia with a multifactorial focus that includes both pharmacological and non-pharmacological interventions [2]. Pharmacological treatments of PWD principally address to cognitive impairment. Cognitive impairment constitutes the most relevant demonstration of dementia and it’s tend to be associated to different symptoms that affect the individual’s functional abilities, which interfere with the habitual activities and reduce their quality of life (QL) and the QL of their caregivers [3-5]. It has been found that some drugs can help delaying the development of behavioral symptoms and that these drugs can contribute in a substantial way to improve the QL of the patients, their relatives, and their caregivers. But it has also been observed that the administering of antipsychotics in PWD can increase the mortality and the risk of cerebrovascular accidents [6]. A recent review [2] concludes that the non-pharmacological therapies (NPT) can be a useful, versatile and potentially cost-effective tool to improve clinical demonstrations and the QL of the PWD, as well as the caregiver. In respect to the cognition results, functionality for the Basic Activities of Daily Living (BADL), behavior and mood, the magnitude of the effect achieved with different NPTs is similar to that observed with the use of drugs. Due to the widespread absence of secondary effects and its flexibility to be adapted to individual cases, the NPTs should be the first choice of therapy to improve the functionality of the BADL or to modify specific behaviors [2,7].

### Non-pharmacological therapies: physical activity

Several randomized clinical trials (RCTs) in PWD have shown that physical activity (PA) is a beneficial intervention for healthy older people, because it may increase the functional capacity, control for cardiovascular risk factors and slow the progression of cardiovascular disease [8,9]. It is emphasized in the reviews on the NPTs in dementia [2,10], that there is a need to carry out major RCTs in the area of physical exercise. In various RCTs with PWD, PA seems to provide benefits for the QL, problematic behaviors, depression, functionality, and falls [11,12], but they do not find evidence that it will improve CVD in the long-term. Nevertheless, recent results are encouraging [13-15] because they suggest a potentially beneficial relationship between physical activity and cognitive function, and they are designing new trials that may help clarify this issue [16-20]. Baker et al. [21] found that there was improvement in the executive control in sedentary women with CVD after six months of high intensity aerobic activity. With the current data, it seems aerobic exercise interventions are more effective than those of stretching [14,16,22]. CVD and dementia are closely related—whether in the form of Alzheimer’s disease (AD) or CVD—and they share very similar risk factors [3,23-25]. It’s possible that there are many mediation mechanisms that improve the CVDin relation to PA, by improving brain vascular function, cerebral perfusion, and the stimulation of synaptogenesis [16]. Reinforcing data from recent years suggests that modifiable behavior can have an impact on brain plasticity in human beings and animals [26]. Although unclear which mechanisms can influence the deposition of an amyloid, it is also possible that exercise can have direct relative effects on the metabolism of glucose and proteins through neurotrophic factors, neuroinflammatory factors, and cerebrovascular functioning [27]. Therefore those interventions that are recommended to improve the CVD may be beneficial to improve the cognitive level, such as walking quickly on flat terrain, at least 30 minutes every day of the week [3,23]. On the other hand, it is unclear why some cognitive functions seem to improve with physical exercise, while other functions seem to be insensitive to the change. This is an aspect that has been challenging to assess since there has been no agreement on the minimum battery of cognitive tests that must be carried out in order to detect clinically relevant and transparent changes, and that allow an increase in the reproducibility of the results in future investigations [12,19,21,25].

### Interventions with people with dementia and with their caregivers

One limitation of the effectiveness of NPT on PWD is the difficulty of carrying them out in the real environment where the PWD and caregivers are [2]. Sometimes it is only possible to do so in specialized centers and almost always is a burden for the caregiver, aggravating the negative consequences that their own care entails, such as the deterioration of the mental health, poor social support and the decrease of leisure time [28]. Of the interventions offered to CG of patients with dementia, the interventions are predominately psychosocial [2,29]. One of the recommendations that should be done is to increase the time they have for themselves, since making pleasant activities is a proven method to improving one’s mood [30]. It is therefore important to identify which activities you wish to participate in and to make a detailed list of the activities that can be increased, noting when they will take place. Pleasant activities are not only extraordinary activities like going on vacation to a far away place. They can actually be performed daily (reading the newspaper, knitting, talking with a friend on the phone, visiting friends, etc.). Additionally it is known that one of the disadvantages that the CG encounters when attempting to carry out rewarding activities is that it can be impossible to separate themselves from the patient while they are carrying out their duties [28]. On the other hand, it has been observed that it is difficult for some caregivers to make the decision to carry out pleasant activities when these activities do not directly affect the improvement of the status of the family member whom they care for [30]. A consequence of this is that the CG of PWD, when compared to non-caregivers, participate in less PA [31], show increased cardiovascular risk [32], have an increased risk of hypertension [33], and suffer more frequently from CVD [34,35]. Although these problems can be associated with the state of chronic stress related to the care, and the difficulty in expressing emotions [36], there is no doubt that they are closely related to PA restriction [30,37]. Von Kanel and col. have observed that caregivers, who reported high levels of PA, had a cardiovascular risk score similar to non-caregivers with the same level of PA [38]. These results suggest that if they increase the levels of PA, the CG could decrease their cardiovascular risk to that of the non-caregivers. It is therefore necessary to evaluate the effectiveness of carrying out interventions that encourage PA for CG of PWD [38]. In addition to assessing the degree PA of the caregivers, it seems necessary to develop interventions that specifically contribute to the increase of their PA. However, it is not easy to find interventions that have managed to increase the PA in adults, which thus would make it possible to reduce the high proportion of sedentary subjects that are in Spain. It is estimated to be 75% [8]. At the community level, a Cochrane review in 2008 concluded that there is no sufficient data to support the hypothesis that the community interventions from the multiple components effectively increase the levels of PA of the population [39]. Some RCTs developed in the field of Primary Health Care have achieved positive results, but this was only seen with the help of PA professionals or educators, and the family doctor. There was an increase of 9.7% in PA for the intervention group with the “green prescription” [40]. Other results have had discrepancies between men and women [41], and if it appears to be effective in the increasing PA of the elderly [42]. Within the framework of the European year 2012, the year of active aging and intergenerational solidarity, the HAPPIER study (Healthy Activity &Physical Programs Innovations in Elderly Residences) was initiated to be developed in elderly residences. However, there is more evidence that all patients with chronic illnesses should be refered to a rehabilitation program that includes an intervention of PA [43]. Since chronic illness is stable, it seems reasonable that it should be managed in Primary Health Care and coordinated as a regular practice. The program has been initiated into the PACE-Lift in UK in order to determine the feasibility and effectiveness of a Primary Health Care intervention with a pedometer to increase PA among older patients [44]. Our research program has participated in the project “Multi-center Evaluation of the Experimental Promotional Program of Physical Activity” (PEPAF) [8], from a sample of 5,000 subjects which were selected randomly from the population consultant in Primary Health Care of six Spanish provinces. The intervention was carried out in Primary Health Care centers and has been effective in increasing the level of PA among inactive patients [8]. Controls for PA were increased to 18 minutes per week [95% 6–31 min/week]; with an increase of the METS/hour week of 1.3 [95% Cl, 0.4 2.2]. But what is most important in relation to this new project is that the effect of the intervention was particularly positive in people older than 50 years [8], therefore it could be appropriately applied to the PWD and their CG. On the other hand, in older adults there seems to be a linear relationship between the level of activity and health outcomes, not only among the sedentary, but also among those who walk more than 12,000 steps per day [45]. Our study incorporates a particular perspective in respect to the gender differences, since more than 70% of the caregivers are women, these usually present a greater osteoarthritis, which makes them less possible to carry out PA [30].

### Evaluation of the therapeutic interventions for dementia

There are many difficulties when assessing the effectiveness of the RCTs done on the PWD, both in general and for Alzheimer’s Disease. Therefore, both the Food and Drug Administration (FDA) and the European Medicines Agency (EMEA) have developed several guides to oversee RCTs in patients with AD. It should be noted that in RCTs on Alzheimer’s Disease drugs the Evaluation Scale of Behavior-Cognitive Section (ADAS-cog) has been used almost unanimously as a measure of cognitive performance [18,46]. It is a test that provides clinically significant information, which is well considered on a conceptual and neuropsychological basis [47]. The use of the ADAS-cog as a fundamental measure of the cognitive state should be permitted to draw comparisons with the results from other NPT or even with the RCTs, taking all this into account as it has been proposed in recent trials [15,18] and that is why this measure is proposed for our intervention. Due to the multidimensional nature of PA, there is no ideal way to measure PA since it occurs in natural contexts in a set of complex leisure and instrumental behaviors. These behaviors are conducted at different levels of intensity, duration, and with different frequencies, both within one day and in different seasons of the year. Also PA involves different parts of the body that make that the measure of PA is more effective in some circumstances than in others. The majority of research for evaluating PA has been mainly in adolescents. It is necessary to carry out research that can provide information about the difficulties of measuring PA in PWD. Self-report questionnaires are the most utilized methods for evaluating PA in research. The simultaneous involvement of the accompanying family member of the PWD during the walks, as well as the completion of a validated questionnaire (7-PAR), should provide important information on the possible relationship between the characteristics of PA and PWD. A very important drawback to evaluating PA in PWD based upon the tests carried out, is related to the large variability observed in terms of the dosage (intensity and duration), modality (aerobic, muscle endurance, etc.) and scope of preparation (at the home of the patient [13,48], in residences or in hospital). In order to quantify the exercise dosage, it is important to use objective instruments to measure in this project, such as digital pedometers. On one hand it is important to know PA habits of the PWD and relatives, and on the other hand the effects of increasing their PA.

### Objectives

The objective of the first phase of this study is to evaluate the effectiveness of a Primary Health Care intervention to increase the PA of PWD and their relative caregivers and to keep the increase of PA for at least one year. The study will also evaluate if any effect is modified by age, gender, or participation as a pair. It will also be estimated the effect of the intervention on the patients in regards to their cognitive status, anthropometric measures, cardiovascular risk factors, overall health and quality of life.

### Hypothesis

There are studies that suggest that increasing PA in both PWD and CG seems to provide health benefits in both groups. However, there are no interventions that have demonstrated their effectiveness to ensure that both groups increase their PA.

Since the implementation of the project PEPAF in Primary Health Care it has been effective in increasing PA in inactive adults [8], especially among those over 50 years. We hope to demonstrate that a physical activity program that is adapted, designed, and applied by Primary Health Care professionals for patients with dementia and relative caregivers (predominantly women), is effective in increasing PA by 20% for PWD and their relative caregivers from Primary Health Care. Since the physical exercise seems to contribute and improve or slow down the cognitive impairment in PWD, we hope to reduce the ADAS-cog in PWD by 3 points. Since CVD and dementia are closely related—whether in the form of vascular cognitive impairment or in the form of Alzheimer’s Disease—and they share similar risk factors, if this program is effective, it may benefit people with dementia as well as relative caregivers, since it could help them maintain and improve their health, in particular the cognitive level of their patients and the cardiovascular problems of their CG which are derived from reduced physical activity due to the CG care tasks.

## Methods/Design

### Design

Clinical trial, multicenter studies randomized, controlled, and randomized into two parallel groups.

### Subjects

#### Study population

**Figure 1:**
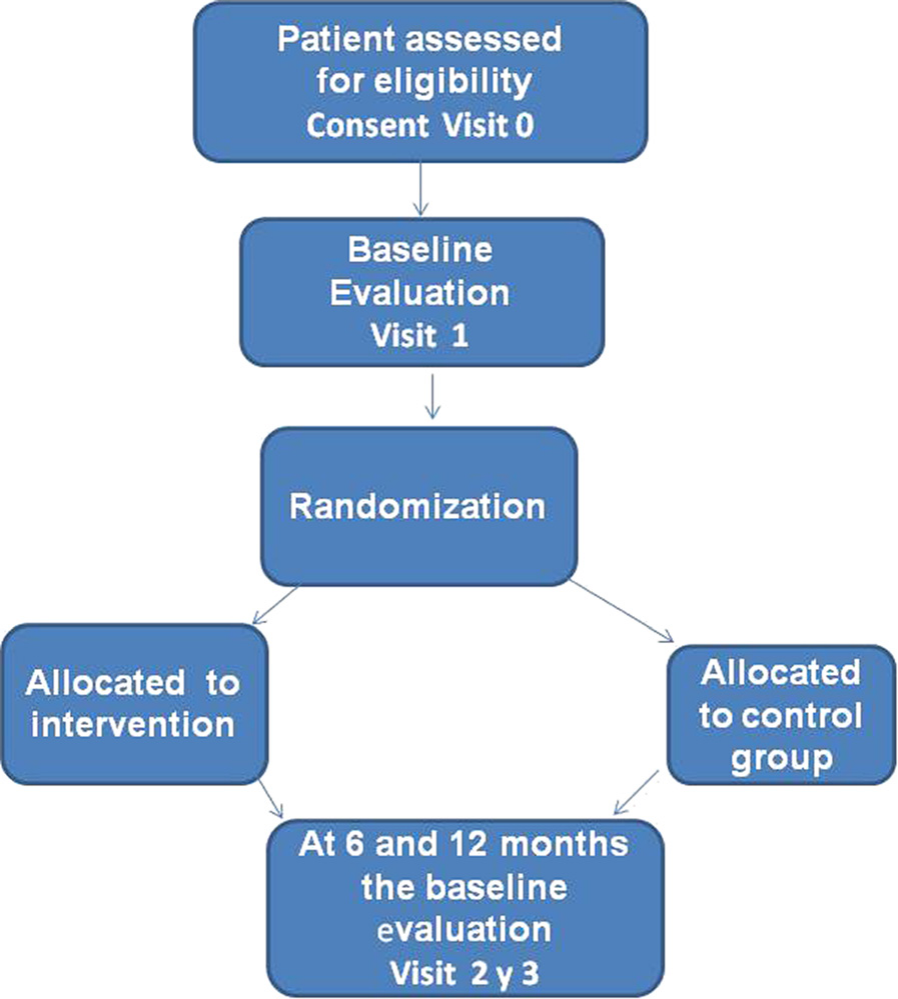
Study outline.

It will be conducted in the scope of Primary Health Care in Salamanca. There will be a simple random sampling from a list of persons who are diagnosed with dementia that belong to the participating centers. Participating physicians will confirm the inclusion criteria and the degree of evolution (GDS-FAST) Global Deterioration Scale of Reisberg. The study will contact caregivers of selected patients who meet the criteria for inclusion and they will be asked to participate in the project (Figure 1). Of those who accept, a list will be created of patients and relatives who provide care for the patient, and at least one person from this list will be selected to participate in the study. If there is more than one family member who cares for the patient at least two days a week, there will be two eligible relatives. Those who accept and meet the criteria for inclusion/exclusion must complete the informed consent document. Then they will be quoted in order to perform the initial evaluation in the Research Unit to both the PWD and the caregiver.

**Inclusion Criteria:** a) patient with dementia that resides in his/her home address in the area of the Primary Health Care center. b) Meets common criteria for the symptomatic diagnosis of dementia DSM-IV confirmed by the family doctor or neurologist. c) Identification of the relatives, or friends (not professional caregiver), who provides most of the care to the PWD. They do not necessarily have to live with the PWD. d) Completion of informed consent on the part of the caregiver (and PWD when appropriate).

**Exclusion Criteria** for the patients or the caregivers: a) mental disorders due to medical illnesses or related to substances (DSM-IV-TR). b) Advanced stages of dementia (GDS 6 or 7), c) Delirium. d) Comorbidity that prevents them from performing the exercise program intervention: unstable heart disease, bedridden, the need for a walker or a wheelchair, significant cerebrovascular diseases such as, bone and muscle disorders, major psychiatric, neurological or metabolic disturbances. e) Severe clinical events within the previous 6 months. f) On the waiting list of surgical interventions or treatments that require a hospital stay, or predict stays out of the capital for more than 4 weeks during the course of the intervention. g) The caregiver dissents to participate in the trial. h) Participants who submit any psychological or general medical condition that in the opinion of the investigator may impede the completion of the questionnaires in the protocol. i) Participants who are taking part at the time of the start of the study in a clinical trial or study involving medications. The PWD take medications that may negatively affect cognitive function: anticholinergics (i.e. amitriptyline), major tranquilizers (antipsychotic typical and atypical) and anticonvulsants. g) Changes in the medication for PWD that they can take for cognitive function (memantine, rivastigmine, donepezil, galantamine) during the period of intervention.

### Sample size

Accepting a 0.05 Alpha risk and a beta risk of 0.2 in a bilateral contrast, it is required to have 67 patients in the control group and 67 in the intervention group (total: 134), to detect a difference equal to or greater than 600 steps/day (1/2 SD) between the 2 groups. It is assumed that the common standard deviation is 1200 steps and it has estimated losses of a 5% follow-up rate.

### Variables and measurement instruments

1. Principle Variable of the Study

It will be an evaluation of PA in the PWD and the CG, and there will be two measurements used:

1.1. The PA carried out during 7 days by the digital Pedometer (Omron Hj-321 lay-UPS) will be used to evaluate the primary endpoint of psychical activity, which had been previously validated [49]. Piezoelectric sensors use multiple-position sensing technology. The manufacturer of the pedometer describes that in this model, an acceleration waveform is generated from both sensors, and based on the size and shape of the waveforms, an algorithm selects the sensor that is used to count steps. The advantage of having two piezoelectric sensors in the pedometer is that it is not critical to position the pedometer perpendicular to the ground and upright at the hip to obtain accurate step counts. For example, if the pedometer is at an angle of 60- or more from the horizontal or at a slant, the pedometer can still accurately estimate steps. It features and displays total steps, aerobic steps, the distance traveled and calories consumed and it stores the results of the last 7 days. Calories burned: By measuring the intensity of your activity, the unit can calculate the amount of calories burned. Aerobic steps: Aerobic steps are counted separately when walking more than 60 steps per minute and more than 10 minutes continuously. If a rest of less than 1 minute is taken after a continuous walk of more than 10 minutes, this will be regarded as part of “a continuous walk”. The PWD and the CG will wear the pedometer, the subject will wear the pedometer on the right side of the waist on an elastic belt for 7 consecutive days. The initial evaluations will be at 6 months and 12 months.

1.2. They will complete a PA questionnaire: The PA is estimated by means of the 7-day Physical Activity Recall (7-PAR). The 7-PAR is a general measure of physical activity, which has been recognized as valid and reliable tool in recent years and is widely used in epidemiological, clinical and behavior change studies. It consists of a semi-structured interview (10-15 minutes) in which participants provide an estimate of the number of hours dedicated to physical or occupational activities requiring at least a moderate effort in the past seven days. The categories collected are moderate, vigorous, and very vigorous physical activity. The amount of time dedicated to each activity is multiplied by the mean metabolic equivalents (METs) of each category: light activity 1.5, moderate 4, vigorous 6, and very vigorous 10. The sum of the products of the hours dedicated to each activity and its estimated mean energy expenditure (MET) provides an estimation of the kilocalories per kilogram used per day (kcal*kg-1 * d-1). The dose of physical exercise will be estimated in METs/hour/week and active persons were considered as those doing at least 30 minutes of moderate activity, five days a week, or at least 20 minutes of vigorous activity, 3 days a week or 450 MET · min · wk^-1^. Persons not reaching this level of physical activity were considered sedentary [50].

2. Physical Activity Log:

2.1 They will be recorded the PA habits of the PWD and their relatives: places, schedules, habitual activities, leisure, instrumental, and sports activities; the adaptations to climatic and to seasonal; covered spaces, outdoor; at home or in institutions. The changes in the family care plan for increasing the PA. There are explanations on the possible discrepancies in registration of the pedometer and the 7-PAR questionnaire.

2.2 Contributions by medical staff and nurses on the implementation of program centers and on the degree of hospitality will be recorded to offer non-pharmacological therapy with the support of scientific evidence.

2.3 The evidence related to the PWD’s use of pedometers will be studied: mode of employment, possible loss, forgetfulness, improper use, etc.

3. Patients with Dementia:

3.1. As the primary measure of cognitive performance, ADAS-Cog will be applied [51,52]. This test is made up of various scales that assess memory, learning and recognition, language, visuoconstructive abilities, ideational praxis, and temporal-spatial orientation. The score is given based upon error and the score can range between 0 and 70 points. Higher scores indicate a greater severity of cognitive impairment. This test has been important for measuring outcomes in numerous AD trials.

3.2. Two cognitive screening tests will be also administered to assess the general cognitive status: a) Mini-Mental State Examination (MMSE). This test [53] explores the functions of temporal-spatial orientation, attention, memory, language and constructional praxis, with a maximum score of 30 points. b) The 7-minute Screen test (7 MS). This test [54] is made up of four subtests: Benton Temporal Orientation Test, Enhanced Cued Recall Test, Clock Drawing Test and Categorical Fluency. It will evaluate the functions of orientation, episodic memory, visuoconstructive abilities and verbal fluency, usually altered in Alzheimer’s disease.

3.3. In addition they will be evaluated based upon the (Reisberg GDS) global deterioration scale, a scale of dementia (Blessed, Timlison, and Roth, 1968) and the ability to develop basic activities of daily living (BADL): Barthel Test and Lawton-Brody Scale.

4. Caregivers: it will be evaluated by the Quality of Life (SF-12), Mental Health (GHQ-12), Family Functioning (APGAR-F), and Overload (Zarit Brief).

5. Other variables of the CG and the PWD: sociodemographic variables (age, sex, education level, household, number of living children, marital status and relationship between patients and CG), anthropometric variables (weight, height, and waist circumference), blood pressure levels and analytical (cholesterol, triglycerides, and blood sugar). The cardiovascular risk (Framingham-D’Agostino) and the Charlson comorbidity index will be calculated. Medications taken that may be linked to cardiovascular disease, cognitive impairment or with mental illnesses will be logged.

6. Characteristics of the PWD and the CG and the motivation to increase the PA by means of the Test Prochaska-Diclemente will be evaluated.

### Baseline evaluation and follow-up

The initial information will be collected in the 1^st^ visit: It’s comprised of 3 groups of assessments (carried out by different professionals): A) The initial (health) screening: diagnostic criteria, GDS, dementia (Blessed, Timlison and Roth, 1968), informed consent, characteristics of care (duration, intensity, social support), informant questionnaire (TIN), socio-demographic data, comorbidity. B) Psychology: a) Patient: Mini Mental State Examination, ADAS-cog, 7-Minute Screen Test. C) Nursing: Cardiovascular evaluation, anthropometric measures, pedometers. b) The caregiver: Quality of Life (SF-12), Overload and burden (Zarit Breve), Mental Health (GHQ-12), Family Functioning (APGAR-F). In VISIT 2, after 7 days, the pedometers and activity log will be collected (7-PAR); It will be confirmed to the Principal Investigator that the initial information is complete and the pair will be assigned to the corresponding group (intervention or control (1:1) according to the previously maid random sequence. At 6 and 12 months the baseline evaluation will be repeated for both the CG and PWD. The flow chart (Figure 1) details the phases and steps in the development of the clinical trial.

Information on the activities carried out, difficulties in performing them, activity log, etc. will be collected during the interviews when the pedometer is collected. After the 6 month evaluation, there will be at least one meeting with the caregivers in the intervention group and another with the control group to complete the information about the development of the program and the proposals that have been considered more efficient to better record the PA (use of pedometers, etc.) and, in the intervention group, contributions are also prompted to increase the PA in their environment.

### Intervention

During the 3 months they will receive instructions to perform PA autonomously (walking, preferably in the vicinity of the place of residence). The intervention will be conducted by a professional from the health center so only pairs from the intervention group will have knowledge of the specific recommendations of the AFISDEMYF. There will be a 15-minute interview each month. This is an intervention that has demonstrated its effectiveness in the PEPAF study [8] and adapted, designed, and been implemented by Primary Health Care professionals for dementia patients and their relative caregivers.

### Assessing allocation concealment

The researcher who will make the randomization will be a different researcher to the one making the assessment. Nursing and psychology professionals whom are not informed about the group that each participant belongs shall make the evaluation visits. The subjects of both groups may participate freely in any other activity during the intervention period and will continue to participate in those that have been previously initiated.

### Timeline

In 2013 the ethics committee approved the study, and the evaluation questionnaires and procedure manuals were drafted. Recruiting of the pairs and baseline assessments will take place in 2014. Every pair will be evaluated again at 6 and 12 months, including the measurements from the initial phase. Starting from the third quarter in 2014, there will be meetings held with successive groups of 10–12 caregivers to complete the information on the difficulties encountered when increasing the PA for the PWD as well as the caregivers and on the difficulties that are observed in order to quantify the amount, intensity and duration of the PA that the PWD performs. In the last stage of the project, a physical activity program guide will be developed for patients with dementia and their relative caregivers, and a technical report on the characteristics and recommendations for designing pedometers that are adapted to the PWD with the aim of facilitating their commercialization and incorporate them into “memory stores”.

### Statistical analysis

The analysis will take place using the intent- to-treat (ITT) The observed improvements will be compared between the basal measurement, at 6 and 12 months for each subject assigned to the intervention groups with respect to those assigned to the control group. The size of the effect will be defined as the change post-treatment in the experimental group less the change post-treatment in the control group, divided by the standard deviation combined. The effect of the intervention will be attributable to the differences in such improvements in these groups. A 95% confidence interval will be calculated. Predictor variables will be analyzed and confounding factors: sex, age, and it will be estimated the time’s effect on the repeated measurements that were carried out, using mixed linear regression models, fixed effects (time, intervention, interaction between time and intervention) and at random. The data was analyzed using IBM® SPSS® v.20 software.

### Quality control

In order to ensure data quality, the professionals in charge of data collection will receive specific training. Regular external monitoring will then be performed in the six health centers to verify adequate application of methods, both in performing the different examinations and collecting the information.

### Methodological limitations

The study follows all the CONSORT recommendations, but due to the nature of the intervention, the participants will not be blinded to the intervention. The work carried out thus far with PWD have not evaluated the modifications on the PA level for PWD or CG, so the comparison we have made based on the results obtained in adults or elderly people. The monitoring of PWD can present specific difficulties for performing PA, which have not been detected in studies of other groups. The effectiveness of an intervention that increases the PA in PWD/CG pairs has also not been evaluated, which can be particularly important, although it is not possible to estimate in which of the two groups (patients or caregivers) there will be a greater impact from the intervention. In regards to the validation of the measurements provided by the pedometer, a proven model of pedometer has been chosen (Omron HJ-321 Tri-Axial) and since the measurement of the outcome considers the changes to the step count before and after the intervention by using the same device, the validation is less conclusive. With respect to secondary outcomes, related to the changes in the cognitive status and cardiovascular risk, it would follow-up period exceeding the duration of the study would be required to obtain clinically relevant conclusions.

### Ethical and legal issues

The Ethics Committee of the University Hospital of Salamanca (Spain) approved the project on April 11, 2013. The subjects will be informed of the project’s objectives, and the risks and benefits of the measures carried out. Prior, they will sign an informed consent in accordance with the Declaration of Helsinki [55]. None of the measures present vital risks to the type of subjects that will be included in the study. The confidentiality of the included subjects is ensured at all times by the Organic Law of Data of Personal Nature (15/1999 of 13 December, LOPD) and under the conditions of the biomedical research of Law 14/2007.

## Discussion

Recent demographic changes have led to the aging of the population, and thereby to the increase of chronic diseases and disability, making a greater commitment of care by family members a requirement. This situation has particularly impacted women, since they represent more than 70% of relative caregivers, so it is necessary to incorporate a gender perspective in the research content for these diseases, an objective that occupies a prominent place in this project.

Dementia and vascular diseases are closely related and share similar risk factors. There are studies that suggest that the increase in PA can bring benefits for PWD and their CG. The recommendation for CG and PWD to participate in therapy at the same time is possibly beneficial for both, and they can receive additional benefits when performed regularly together, than if it is performed separately. As a non-pharmacological intervention, it has the potential to be an effective, efficient and accessible strategy that can be conducted especially in the field of Primary Health Care and which could be applied even in areas with scarce resources, since the intervention requires a low cost. Responsibility is thus in the hands of institutions that have sufficient funds to ensure the development of research and dissemination of the effectiveness of this therapy. The completion of a validated questionnaire and measure of contributing pedometers, should provide important information on the possible relationship between the characteristics of the PA held (in terms of dose, modality and scope of preparation) and the observed changes. Since it is known that the availability of pedometers has managed to improve interventions aimed at increasing PA, it seems advisable to investigate the characteristics of these devices in this group in particular. Current expectations of increasing the PWD, with the raise of PA between leisure activities as well as therapies, are important incentives that can be offered to companies of both sectors. These recommendations could be derived from this study. It is without a doubt that this is an extraordinary opportunity to apply scientific knowledge to improve the prevention, diagnosis, and treatment of the sedentary lifestyle, helping to improve the current health indicators of cardiovascular disease and dementia.

It is important to know, on one hand, habits of PA of PWD and their relatives, and on the other hand the effect of increasing their PA. In the third phase the study intends to evaluate the indirect effects of the program on the welcoming and establishment of Primary Health Care Centers. It will try to measure the contribution of the program on the relatives of PWD and on the medical and nursing staff (may offer a non-pharmacological therapy with an endorsement of scientific evidence). Others can identify and take advantage of the project’s strengths as to develop PA measurement systems in the groups to which the study adressess and to promote initiatives that reduce scientific and technical difficulties that are detected during the development of the same one.

If the main hypothesis of the study is confirmed, the first international evidence about the effectiveness of the intervention in Primary Health Care to increase PA in PWD/CG pairs will be available. Therefore, a specific guide for PA could be edited and the possibility to collaborate in validating recommended devices will be provided, which will allow to obtain additional funding for the development of new projects. To do so, ‘it will be madea registry of the intellectual property and a patentable study of the developed tool and in collaboration with a technology company they will begin the procedures through OTRI office. Finally there will be a contract/agreement with the developer company for its marketing and generating funds for future joint projects.

The development of the research project will promote public-private coordination through the coordination of the Primary Health Care network, which can enhance the research in the development of technologies to measure the PA. If the expected results were achieved, the incentive that enterprises require to invest in this program would rely on the possibility of being supported by scientific evidence because of the importance of relevant health issues that present the targeted groups (dementia and cardiovascular diseases) and also having the possibility of introducing the use of pedometers through the largest health care network such as Primary Health Care.

### Trial status

The trial is currently in the recruitment phase.

## Abbreviations

AD: Alzheimer disease; ADAS-cog: Alzheimer’s disease assessment scale-cognitive; BADL: Basic activities of daily living; CG: Caregivers; CI: Cognitive impairment; CVD: Cardiovascular disease; NPT: Non-pharmacological therapies; PA: Physical activity; PWD: People with dementia; QL: Quality of life.

## Competing interests

The authors declare that they have no competing interests.

## Authors’ contributions

Conception of the idea for the study: ERS, SMS, LGO, JMCG and MAGM. Development of the protocol, organization and funding: ERS, SMS, MPMD, JIRR, MCPA 1,4, LFVJ 1,5, and JAMF. Writing of the manuscript: ERS, LGO and MAGM. All authors read and approved the final manuscript.

## Pre-publication history

The pre-publication history for this paper can be accessed here:

http://www.biomedcentral.com/1471-2377/14/63/prepub

## Participating centers

**Primary care research unit of La Alamedilla Health Center**, (Health Service of Castilla y León): Luis García-Ortiz, Manuel A Gómez-Marcos, Emiliano Rodríguez Sánchez, José I Recio-Rodríguez, Sara Mora Simón, Jaime Unzueta Arce, C. M Carmen Patino-Alonso, Cristina Agudo Conde, José A. Iglesias Valiente, Diana Pérez-Arechaederra, José A. Maderuelo-Fernández. M Paz Muriel-Diaz and Luis F Valero-Juan.

**Department of Basic Psychology, Psychobiology and Behavioral Sciences Methodology, Faculty of Psychology,** University of Salamanca, Salamanca (Spain): M. Victoria Perea-Bartolomé, Sara Mora-Simón, Valentina Ladera-Fernández, Ricardo García-García, Jaime Unzueta-Arce and Emiliano Rodríguez-Sánchez.

### DERIVA Group

Research about DEmentia and cardio VAscular RIsk.
